# The effect of basic psychological needs on the flow experience in a digital gamified learning setting

**DOI:** 10.3389/fpsyg.2023.1256350

**Published:** 2023-09-15

**Authors:** Sarah Lüking, Sarah Wünsche, Matthias Wilde

**Affiliations:** Biology Didactics, Faculty of Biology, Bielefeld University, Bielefeld, Germany

**Keywords:** motivation, basic psychological needs, digitalization, gamification, flow, flow experience, biology education

## Abstract

**Introduction:**

Digitalization and gamification offer numerous motivation-enhancing opportunities to design biology lessons. For example, digital, gamified learning settings can enhance lessons by offering intense experiences. Such lessons might offer the opportunity to witness flow during the learning activity. For learners, flow can be positively influenced by perceived autonomy, competence, and relatedness. However, previous research on biology lessons has not focused on the impacts of the basic need satisfaction on the flow experience in digital learning settings.

**Methods:**

To address this research gap, using the topic of the locomotion systems of animals, we investigated students’ perceived autonomy, competence, and relatedness as possible predictors of their flow experience while processing a digital, gamified learning environment. The teaching unit was thematically focused on the locomotion system of animals. Our sample consisted of 161 students (46.6% female) from sixth to eighth grade. Students’ perceived satisfaction of their basic needs and their flow experience were evaluated.

**Results and Discussion:**

Results confirmed perceived autonomy and perceived competence as predictors of students’ flow experience. However, perceived relatedness had no impact on the flow experience. Our findings are in line with the current state of research and prove to be mostly consistent with previous results.

## 1. Introduction

Positive motivational qualities are important criteria to promote positive learning experiences in school and foster academic success (Ruppert, 2013; Ryan and Deci, 2017). An adequate degree of self-determination, tasks that match the students’ abilities, and a stimulating learning environment can have a positive effect on motivational qualities (Ryan and Moller, 2016). In intrinsically motivated learning, the driving force lies in the action of learning itself, which becomes the goal of learner behavior (Deci and Ryan, 1985). A rather autotelic aspect of intrinsic motivation is flow experience. In a flow state, the complete absorption in an activity is experienced. Action and awareness merge and an absorbedness in the task occurs (Nakamura and Csikszentmihalyi, 2014). An activity should fulfill certain requirements in order to foster flow experience: (a) the structure of a task needs to be very clear; (b) the learner receives unambiguous feedback on their performance and knows at all times what to do; (c) and there is no room for misunderstandings (Nakamura and Csikszentmihalyi, 2014; Engeser and Schiepe-Tiska, 2021). Furthermore, the task should present an optimal challenge by creating a balance between the requirements of the task and the skills of an individual (Csikszentmihalyi, 2000).

Digital gaming elements might fulfill these requirements. By adopting digital gaming elements into biology lessons, the flow experience could be supported in the learning process. Both, intrinsic motivation according to Ryan and Deci’s self-determination theory (Ryan and Deci, 2017, 2020) and flow theory according to Csikszentmihalyi (1975; see Nakamura and Csikszentmihalyi, 2014) describe an autotelic situation. During an autotelic experience, the action is performed regardless of external consequences. External stimuli are not involved, since the experience of the action itself is the reason to act (Nakamura and Csikszentmihalyi, 2014). Flow experience is often intertwined with the occurrence and development of intrinsic motivation. Both flow experience and intrinsic motivation have been found to positively affect school performance (Csikszentmihalyi and Schiefele, 1993; Kowal and Fortier, 1999). The relationship between flow experience, intrinsic motivation, and basic need satisfaction has been investigated in previous studies (Kowal and Fortier, 1999; Lüking et al., 2021; Wilde, 2021). The satisfaction of the needs for autonomy and the need for competence have already been identified as predictors for the flow experience in out-of-school learning settings (Lüking et al., 2021). In these settings, learners can directly interact with original objects by touching or smelling them, and their actions may merge with the environment to a high degree. In digital learning settings, information processing is limited to two sensory channels, the visual and auditory. In addition, there is a difference in the possibilities of communication with classmates. These are rarely addressed in digital learning environments, because they are highly individualized (Hillmayr et al., 2020). In the field of biology education, there is currently no empirical evidence that the perceived satisfaction of basic needs is related to the flow experience in digital learning settings. The following study aims to address this research gap.

## 2. Theory

### 2.1. Self-determination theory of motivation

Motivation is a very relevant factor in educational settings. Self-determination theory (SDT) is a framework to describe, understand, and explain human motivation (Deci and Ryan, 1985, 2000; Ryan and Deci, 2000a,b, 2017, 2019, 2020, 2023). As Ryan and Deci (2000b) point out, motivation requires intentional activities: “To be motivated means to be moved to do something” (p. 54). SDT distinguishes between intrinsic and extrinsic motivation (Ryan and Deci, 2000a,b, 2017, 2020). Intrinsic motivation is characterized by self-determined pleasurable, proactive, and engaged behavior (Ryan and Deci, 2000a). These activities are inherently interesting and enjoyable (Reeve, 2015). By contrast, extrinsically motivated actions can be categorized by their instrumental intention (Deci and Ryan, 2000; Ryan and Deci, 2020). Extrinsic motivation refers to doing something because it leads to a separable outcome (Ryan and Deci, 2000b, 2017). Still, the quality of extrinsic motivation might differ substantially; for example, a student could perform an extrinsically motivated action with resentment and resistance or with an attitude of willingness and with acceptance of the utility of the task at hand (Ryan and Deci, 2000b, 2017). Depending on the level of perceived heteronomy or perceived autonomy, respectively (Vansteenkiste et al., 2011), a more or less passive and alienated type of extrinsic motivation can be hypothesized. Deci and Ryan (2000) and Ryan and Deci (2000a,b, 2017, 2020) describe different regulatory styles ranging from external, introjected, identified to the integrated type of regulation. In the following, the two most pronounced regulatory styles within extrinsic motivation are described, external and integrated. Regarding external regulation, a behavior is performed to satisfy an external demand or an externally imposed reward (Ryan and Deci, 2000b, 2017, 2020). A student might feel controlled or alienated from the task and perceive his/her action to have an external locus of causality (De Charms, 1968). This type of regulation reflects the most heteronomous form of extrinsic motivation. On the other hand, in integrated regulation the student still performs the action for an instrumental reason but with an attitude of willingness and inner acceptance of the value and utility of a task (Ryan and Deci, 2000b). Hence, it reflects the most autonomous form of extrinsic motivation (Ryan and Deci, 2000a,b, 2017, 2020). As educational activities at school are not usually designed to be intrinsically interesting, the processes that might transform behavioral regulations from perceived heteronomy to perceived autonomy play a key role (Ryan and Deci, 2000b; Vansteenkiste et al., 2011). For SDT, these are the processes of internalization and integration of values and behavioral regulations (Deci and Ryan, 1985). Internalization is characterized by taking in values; furthermore, integration entails the transforming of values and regulations into the persons’ self. The result of this process of integration might be a student’s behavior of active personal commitment (Ryan and Deci, 2000b, 2020).

Intrinsically motivated behavior and further positive qualities in motivation are nurtured by the satisfaction of three innate basic psychological needs: autonomy, competence, and relatedness (Ryan and Deci, 2000b, 2017; Reeve, 2015). In SDT, these basic needs energize one to engage in new tasks and to exhibit a proactive willingness to explore and to seek challenges; well-being and growth are associated with the satisfaction of autonomy, competence, and relatedness (Ryan and Deci, 2019). The perception of autonomy describes a congruence of the self of a person and his or her behaviors (Ryan and Deci, 2019). The locus of causality of an action lies within the self of the person, meaning he/she feels like he/she is the “origin” (De Charms, 1968) of a volitional behavior and thus does not feel alienated from it (Ryan and Deci, 2000a, 2017). Meaningful choices may foster the perceived satisfaction of autonomy (Katz and Assor, 2007). In SDT, competence is the psychological basic need that is required to be effective in interactions with the environment and to extend one’s capacities and skills (Reeve, 2015). A feeling of competence might arise when a person is optimally challenged, that is, there is a high degree of congruency between skills and competence and task demand; the challenges are appropriate to develop and to improve one’s capacities (Deci and Ryan, 1985; Reeve, 2015). The need for relatedness that describes the desire to be emotionally and meaningfully connected to other human beings, to be involved in warm relationships, to care and to feel cared for (Baumeister and Leary, 1995; Reeve, 2015). In formal education, the classroom climate might influence the perceived relatedness of the students (Anderman, 2003; Maxwell et al., 2017). Intrinsic motivation can be predicted by the satisfaction of these basic needs (Niemiec and Ryan, 2009). In the school environment, the teacher’s support of basic needs satisfaction can facilitate students’ autonomous self-regulation for learning, academic performance, and wellbeing (Niemiec and Ryan, 2009). In addition to basic need satisfaction and intrinsic motivation, the experience of flow also has a positive effect on students’ outcomes at school (Engeser and Vollmeyer, 2005). Flow represents a complementary perspective on intrinsically motivated actions (Csikszentmihalyi, 1975, 2000; Nakamura and Csikszentmihalyi, 2014).

### 2.2. Flow theory

A particular autotelic type of intrinsic motivation is the experience of flow. This is an autotelic state in which the action is performed entirely without external stimuli. The reward is in the action itself, so that the action itself becomes the goal (Nakamura and Csikszentmihalyi, 2014; Ryan and Deci, 2017, 2020). Nonetheless, an external stimulus may lead to the action and still flow might be experienced in the action (Csikszentmihalyi, 2000). To achieve the flow state, some requirements have to be accomplished. First, the demand of the challenge has to match the skills of the acting person. If a challenge places excessive demands or one’s own skills are insufficient, an anxious feeling or fear can occur. By contrast, if the skills of the acting person are too high or the requirements of the challenge are too low, boredom can occur (Csikszentmihalyi, 1987, 2000). Furthermore, goals need to be clearly structured, and one needs to receive unambiguous feedback on his/her performance (Nakamura and Csikszentmihalyi, 2014). Certain components characterize the flow experience (Csikszentmihalyi, 1975, 1987, 2000; Rheinberg, 2008). Learners are completely involved in their activity and accomplish their task literally in one flow; it is carried out according to its own inner logic, is coherent, and contains no contradictions (Rheinberg et al., 2003). The learners focus their undivided attention on the task at hand, for example they exhibit a very high degree of concentration. Further, their sense of time is affected considerably, as hours might seem to last only moments. A person in a flow state does not reflect on him-or herself, for the self and the activity are not separated. In particular, if a task offers an optimal level of challenge to the learner, flow might evolve (Csikszentmihalyi and Schiefele, 1993; Reeve, 2015). Furthermore, flow provides an autotelic experience. Even if the initial stimulus for the action might be external, the performance of the action is perceived to be intrinsically motivated. The action itself becomes the goal. The relationship between the autotelic state, of intrinsic motivation and flow experience, and its positive impact on school performance has already been investigated (Csikszentmihalyi and Schiefele, 1993).

### 2.3. Digitalization and gamification in schools

As digital technologies become distinct features of our society, the number of digital learning environments is constantly increasing (Peters, 2000; Fraillon et al., 2014; Hodges et al., 2020; Seufert et al., 2021). The goal of schools is to prepare students to use digital technologies responsibly (Sailer et al., 2021). Various studies have indicated positive effects of using digital media in the classroom (Kapp, 2012; Hillmayr et al., 2020). According to these studies, learners feel more comfortable and motivated in digital learning environments. In addition, the use of digital media also enables learning content to be taught more effectively, especially in the areas of natural sciences and mathematics (Hillmayr et al., 2020). Gamification is one possibility to design lessons digitally. The term gamification is used when game elements, such as scoring, quizzes, or puzzles, occur in a non-game context that does not exclusively address entertainment as in games (Deterding et al., 2011). Using game design elements in a non-gaming context might increase engagement and motivation and changes in behavior might be promoted (Deterding et al., 2011; Kapp, 2012). Gamification enables the use of individual elements of a game without needing to use or play an entire one (Deterding et al., 2011). In gamified learning environments, learners might perceive enjoyment and satisfaction when engaging with gamified activities given they follow defined goals and receive clear feedback (Bai et al., 2020). By using gamification elements, learners’ intrinsic motivation can be fostered while giving them the opportunity to satisfy their basic psychological needs (Sailer et al., 2017; Sotos-Martínez et al., 2022). According to Sailer et al. (2017), certain game design elements may be linked to the psychological needs of the learner. Freedom of choice and storytelling address the need for autonomy. When choosing an avatar or a background story, the player perceives his own actions as meaningful (Rigby and Ryan, 2011). The need for competence can be satisfied through scoring points. This gives the students immediate feedback on his or her actions (Sailer et al., 2017). The need for relatedness can also be satisfied through the narrative frame of the game and the perceived meaningfulness of one’s own actions. In addition, this need might be addressed while playing together with teammates (Rigby and Ryan, 2011). Since flow often arises during games (Engeser and Schiepe-Tiska, 2021), basic needs satisfaction and flow should be investigated in a digital, gamified learning setting.

## 3. Research question

In addition to intrinsic motivation, the satisfaction of basic needs may also foster the level of flow experience (Kowal and Fortier, 1999; Schüler et al., 2013). In biology education in traditional classroom settings and in out-of-school learning settings, there is already some evidence that the satisfaction of basic needs and the experience of flow may be positively related (Großmann and Wilde, 2016; Lüking et al., 2021). These findings lead to the question of whether the relationship between basic need satisfaction and flow experience can also be transferred to digital learning settings. Gamification might promote motivation through the use of elements from entertainment games in the design of digital learning settings (Werbach et al., 2012; Dicheva et al., 2015; Sotos-Martínez et al., 2022). Game design elements include avatars, a narrative context, feedback, and scoring points (Sailer et al., 2017). When using these, the basic needs for autonomy, competence, and relatedness might be addressed and might increase the learner’s autotelic qualities of motivation (Schüler et al., 2013; Sailer et al., 2017). This raises the question: Do the three basic psychological needs for autonomy, competence, and relatedness represent predictors for the flow experience of students in the digital gamified learning setting?

## 4. Methods

### 4.1. Sample

Our sample included 161 students (46.6% female) from four schools (56.5% Gymnasium, 43.5% Gesamtschule) in the state of North Rhine-Westphalia (Germany). The students were in the sixth to eighth grades (6th grade = 42.2%, 7th grade = 42.2%, 8th grade = 15.5%). The average age was 12.7 years.

### 4.2. The digital learning program

Using the topic “The World of Movement,” the digital learning setting was developed by students at Bielefeld University as part of a course for prospective biology teachers. The aim was to promote the university students’ digital skills by introducing them to a way of developing a digital learning setting themselves based on the theory of gamification. After a short introduction to H5P, they designed four digital series of workstations addressing students between the age of 12 to 14. In an iterative process the university students designed the digital learning setting. The result was a website that could be exclusively entered by the students who conducted our study. On the website, learners could gain knowledge on different topics related to the locomotion system of animals. To introduce the learners to the website, the avatars of two researchers were introduced. The learners could choose between a male or female scientist as their avatar. The avatars served as a guide (Schöbel and Janson, 2018). This gives the students a structure in the digital learning setting, even though the teachers are oftentimes absent (Zeifman and Hazan, 2008). In the process, various animal mascots introduced different subtopics. Using the mascots in the first-person perspective might help the students to better understand and emotionally grasp the situation and possibly arising distractions (Bätz et al., 2007). One of the mascots was Zeus, a Zebra Finch. Zeus first introduced his bird species and then addressed various topics such as the skeleton of a bird and how it differs from humans. Furthermore, he explained how birds fly and how it is possible for some mammals to fly. The learners accompanied Zeus with their own avatar through this topic and repeatedly received questions, information, or explanations. The information was presented using text, videos, or illustrations. Direct feedback on the answers was given to the learners by the students, so they could score points. Each student used their own computer and completed the digital learning setting on their own, so no conversation or interaction with other students was required. Study Design and Study Procedure.

The teaching unit was conducted by pre-service biology education students in advanced semesters who were not previously acquainted with the learners. At the beginning, the website was introduced and explained to the learners. Then they had time to work independently in the digital learning setting. The study was conducted in the schools, partly in the computer lab and partly with tablets in the classrooms. While doing their independent work, their flow experience was assessed using the Flow Short Scale (Rheinberg et al., 2003). Immediately after working in the digital learning setting, the satisfaction of their basic needs for autonomy, competence, and relatedness was measured using the Basic Need Scale (Van den Broeck et al., 2010).

### 4.3. Measuring instruments

#### 4.3.1. Basic needs scale

A translation of Van den Broeck et al. (2010) basic needs scale to was used to determine the satisfaction of the learners’ basic psychological needs. This scale was assessed with a five-point rating scale with the response options “not at all true” (0) to “completely true” (4). The scale consisted of three subscales: “autonomy” (6 items), “competence” (4 items), and “relatedness” (8 items). Cronbach’s alpha ranging from α = 0.75 to α = 0.81 indicated a satisfactory internal consistency of the subscales (Table 1).

**Table 1:** Measuring instruments used: presentation with one example item each and the reliability (Cronbach’s alpha = α) of the individual (sub-)scales.

Measuring instrument	Example item (number of items)	α
Flow short scale^1^	“I am completely absorbed in what I am doing at the moment.” (10)	0.83
Basic need scale^2^		
autonomy	“I feel that I can be myself in class.” (6)	0.77
competence	“I am good at things I do in class.” (4)	0.75
relatedness	“In class I feel part of the group.” (8)	0.81

^1^Rheinberg et al. (2003); ^2^ van den Broeck et al. (2010).

#### 4.3.2. Flow short scale

The flow experience was assessed using the flow short scale (Rheinberg et al., 2003). The flow short scale (FKS) comprises 10 items, which were also assessed with a five-point rating scale from “not at all true” (0) to “completely true” (4). Cronbach’s alpha was α = 0.83 (Table 1).

### 4.4. Statistical analysis

To assess whether satisfaction or frustration of the basic needs for autonomy, competence, and relatedness are predictors of flow experience, we performed a multiple linear regression in SPSS. The requirements for the multiple linear regression, such as normal distribution, homoscedasticity and homogeneity of variance (Field, 2018) were met.

## 5. Results

This study investigated whether there is a relation between the perceived satisfaction of basic needs and the experience of flow in a digital learning environment. The assessed values for flow and basic needs for autonomy, competence, and relatedness as well as correlations between these factors are shown in Table 2.

**Table 2:** Descriptive statistics (mean, standard deviation) and bivariate correlation coefficients.

	*M*	*SD*	1	2	3	4
flow	2.50	0.62	-			
autonomy	2.36	0.71	0.42**	-		
competence	2.41	0.68	0.41**	0.59**	-	
relatedness	2.86	0.71	0.30**	0.31**	0.34**	-

N = 161; five-point rating scale from 0 = ‘not at all true’ to 4 = ‘completely true’; ** *p* < 0.01.

Based on these values, a multiple linear regression was performed. This resulted in a significant model for the relationship between the experience of flow as the dependent variable and the basic needs of autonomy, competence, and relatedness as independent variables, *R*^2^ = 0.24, corr. *R*^2^ = 0.22, *F*(3,157) = 16.37, *p* < 0.001.

The regression model showed a significant predictor effect for the basic need of autonomy on the learners’ flow experience with a stand. β = 0.25 (*t* = 2.86, *p* = 0.005). For competence, we found a significant predictor effect with a stand. β = 0.22 (*t* = 2.48, *p* = 0.014) for experienced flow. The predictor effect of the basic need for relatedness on students’ flow experience was not significant with a stand. β = 0.15 (*t* = 1.97, *p* = 0.050). Overall, the model indicated that the basic needs of autonomy and competence in particular are variables that predict flow experience when working in a digital gamified learning setting.

## 6. Discussion

The purpose of this study was to determine whether the perceived basic needs of the self-determination theory autonomy, competence, and relatedness are predictors for flow experience in a digital gamified learning environment. We found that perceived autonomy and perceived competence were predictors for flow. However, perceived relatedness did not predict flow experience. It has already been shown that the use of game design elements in a school context can contribute to the satisfaction of basic needs and thus to increased intrinsic motivation (Sailer et al., 2017).

The basic need for autonomy can be addressed by providing meaningful choices (Schüler et al., 2013). This perception of freedom of choice was enabled for the students with regard to the choice of the avatar in the gamified learning setting. In addition, the learners had the opportunity to decide individually when to work on which topic. The provision of these choices may have encouraged them to learn at their own pace according to their preferences, thus achieving an optimal match between the requirements of a task and learners’ individual skills (Rheinberg et al., 2003). This optimization of task demand is an important prerequisite for flow (Rheinberg, 2000).

The need for competence describes the striving to perceive his/her own actions as effective and to expand his/her skills (Reeve, 2015). This is also important for a smooth, automatic flow of an activity. A person who feels competent knows what to do and feels like he/she can control his/her actions (Ryan and Deci, 2017). This, in turn, is a characteristic of the flow experience (Rheinberg et al., 2003; Nakamura and Csikszentmihalyi, 2014). In the digital learning environment, perceived competence was to be addressed via some interactive elements. Through self-assessment tests and a scoring system, the learners always received direct feedback on their actions. These design elements might lead learners to perceive themselves as initiators and their actions as meaningful in order to satisfy the basic need for competence (Ryan and Deci, 2017).

In this study, relatedness was not found to be a significant predictor of flow experience. There may be several reasons for this result. First, a statistical issue needs to be addressed: a β-error cannot be excluded (Cohen, 1982). Hence, further studies should assess the relationship between basic need satisfaction and flow experience. Furthermore, the digital gamified learning setting that was used did not require interaction between students within the learning setting. In order to avoid a competitive situation, the website was designed without sharing the information about the learners’ results within the class.

The results are in line with previous studies (Großmann and Wilde, 2016; Lüking et al., 2021). Großmann and Wilde (2016) investigated the connection between autonomy and flow experience in the school learning setting and were able to show that autonomy promotion can foster flow experience in the classroom. Similar results were observed in a field trip in an out-of-school learning setting. While the needs for autonomy and competence were shown to be predictors of the flow experience, in this study perceived relatedness did not predict flow experience either (Lüking et al., 2021). Lüking et al. (2021) determined a variance explanation of the flow experience of *R^2^* = 0.52. According to Cohen (1988), this corresponds to a high variance resolution; the variance resolution in the present study was medium with a coefficient of determination of corrected *R^2^* = 0.22. It is therefore reasonable to assume that flow experience in a digital gamified learning setting is dependent on additional predictors.

## 7. Limitations, outlook for further research, and conclusion

In general, the basic needs for autonomy and competence from Ryan and Deci’s (2017, 2020) self-determination theory were found to be predictors for flow experience according to Csikszentmihalyi (1975). However, one should note that the extent to which the measured differences in the satisfaction of basic needs might be attributed to the digital gamified learning setting was not captured by this data. Moreover, a corresponding control group in a non-digital setting was not included in this study. In order to better evaluate the predictor effect of the basic needs on the flow experience, further studies should address these issues. For example, certain interactive elements can be beneficial while others can be detrimental. In order to be able to make clear recommendations for action, there is a need for both qualitative studies on the processes behind learning in such digital learning settings and also quantitative studies that examine individual elements of these learning settings. In addition, different digital learning settings need to be investigated. This is because the results found here cannot be generalized for all forms of digital learning settings and therefore only apply to the digital gamified learning setting with the topic of “The World of Movement” that was investigated in this study.

Still, more reliable statements about specific elements of the learning setting and the basic needs as well as the magnitude of the predictor effects of the basic needs on the flow experience could be achieved. A possible confound may be rooted in the novelty effect (Orion, 1989, 1993). This effect refers to the novelty of an unfamiliar learning setting. This can unsettle learners and thus divert their attention from the learning object (Falk et al., 1978). The novelty effect can be circumvented by designing further studies in such a way that digital learning settings are integrated into the lessons several times. The novelty effect could thus be eliminated over several measurements over time. Another, somewhat less time-consuming way of estimating this effect is by surveying how frequently and regularly digital learning settings are used in the learners’ regular lessons.

Nevertheless, it is worthwhile for developers of digital learning settings to consider the connection between the basic needs of autonomy and competence and the experience of flow. This relationship seems to play a significant role in school, out-of-school, and digital learning settings (Großmann and Wilde, 2016; Lüking et al., 2021). Flow experience is associated with high performance quality, that is, good learning success (Bätz et al., 2009), especially since there is already some evidence of improved learning success through the promotion of basic needs in the school learning environment. This leads to the goal of also considering the promotion of basic needs in digital learning settings. The exact way in which these can be promoted to a higher degree in digital learning settings should be the goal of further research.

## Data availability statement

The original contributions presented in the study are included in the article/supplementary materials, further inquiries can be directed to the corresponding author.

## Ethics statement

The studies involving humans were approved by Ethics Committee of Bielefeld University. The studies were conducted in accordance with the local legislation and institutional requirements. Written informed consent for participation in this study was provided by the participants’ legal guardians/next of kin.

## Author contributions

SL: Conceptualization, Formal analysis, Investigation, Methodology, Writing – original draft. SW: Formal analysis, Writing – original draft, Writing – review & editing. MW: Conceptualization, Data curation, Investigation, Methodology, Project administration, Resources, Supervision, Validation, Writing – original draft, Writing – review & editing.

## Funding

The author(s) declare that no financial support was received for the research, authorship, and/or publication of this article.

## Conflict of interest

The authors declare that the research was conducted in the absence of any commercial or financial relationships that could be construed as a potential conflict of interest.

## Publisher’s note

All claims expressed in this article are solely those of the authors and do not necessarily represent those of their affiliated organizations, or those of the publisher, the editors and the reviewers. Any product that may be evaluated in this article, or claim that may be made by its manufacturer, is not guaranteed or endorsed by the publisher.
